# Taking on the Invisible Third Shift: The Unequal Division of Cognitive Labor and Women’s Work Outcomes

**DOI:** 10.1177/03616843251330284

**Published:** 2025-04-21

**Authors:** Anja Krstić, Winny Shen, Christianne T. Varty, Janice Y. Lam, Ivona Hideg

**Affiliations:** 1 177432 Faculty of Liberal Arts & Professional Studies, 7991 York University, Toronto, ON, Canada; 2 67130 Schulich School of Business, 7991 York University, Toronto, ON, Canada; 3 648904 Canadian Women & Sport, Toronto, ON, Canada; 4 Saïd Business School, 6396 University of Oxford, Oxford, UK

**Keywords:** cognitive labor, division of labor, gender equity, subjective wellbeing, turnover intentions, career resilience, COVID-19

## Abstract

The current research focuses on the gendered work-related impacts of the division of unpaid labor. Drawing upon the literature on gender roles and conservation of resources theory, we argue that women (vs. men) are particularly drained due to undertaking a greater proportion of cognitive labor—a hidden form of unpaid labor involved in managing a household—leading to undermined work outcomes. Data were collected weekly (during the early phases of the COVID-19 pandemic) for 7 weeks in April to May 2020 (*N* = 263) and aggregated. Using multilevel structural equation modeling, we found that women (vs. men) reported engaging in a disproportionate amount of cognitive labor in their households, which increased their emotional exhaustion and, in turn, was related to greater turnover intentions and lower career resilience. However, for mothers (vs. fathers), emotional exhaustion and undermined work-related outcomes were driven by disproportionate responsibility for childcare. Hence, division of cognitive labor uniquely affected work-related outcomes of women without children, whereas division of childcare shaped the work-related outcomes of women with children. Overall, this research highlights the differential challenges faced by working women with and without children and the need for gender equity initiatives focusing on both women with and without children.

Scholars have argued that the unequal division of labor within heterosexual, dual-earner households, whereby women continue to take on greater responsibility for the “second shift” of unpaid labor (e.g., housework and childcare), is a major barrier to workplace gender equity (e.g., Poeschl, 2008). By most accounts, the COVID-19 pandemic exacerbated these inequities (McKinsey & Company, 2020) and highlighted that women also often disproportionately take on an additional, less visible form of unpaid labor (Rodsky, 2020)—cognitive labor. Cognitive labor consists of anticipating household needs, identifying options to fulfill these needs, choosing between options, and monitoring whether needs have been successfully met (Daminger, 2019). Ultimately, this increased labor may exhaust working women and lead them to contemplate dropping out of the workforce or downshifting their careers to cope.

Little is known about whether and how engaging in a disproportionate amount of unpaid labor, including cognitive labor, within one's household may spill over to impact work outcomes. This is important to examine because women are more likely than men to engage in all forms of unpaid labor (for a review, see Shockley et al., 2025). In turn, although it has been established that women's greater responsibility for unpaid labor can detract from the quantity of their paid labor (i.e., work hours; Ruppanner et al., 2019), what remains less clear is the impact that unpaid labor may have women's work attitudes and intentions. Further, it is unclear whether the unequal division of cognitive labor will operate in the same manner as that of household labor or childcare in affecting women's work. More specifically, the mental nature of unpaid labor means that it may not detract from work time in the same way as the other forms of unpaid labor. Overall, a deeper understanding of these processes may elucidate additional ways in which women's greater care burden sustains workplace gender inequity.

In this article, we investigated whether and how the unequal division of cognitive labor within households affects work outcomes. Integrating literature on gender roles (i.e., psychological and sociological perspectives on gender; e.g., Eagly, 1987; West & Zimmerman, 1987) and conservation of resources theory (Hobfoll, 1988), we posit that the burden of mentally managing household demands—*cognitive labor*—falls more upon women than men. This is because it is an activity oriented toward others (Reich-Stiebert et al., 2023), and women are typically socialized and expected to be concerned with the welfare of others (Doucet, 2006). We further suggest that cognitive labor is emotionally exhausting because it drains limited resources (DeGroot & Vik, 2020). In turn, emotional exhaustion is related to efforts to mitigate resource loss, thereby promoting turnover intentions and a diminished capacity to cope, leading to undermined career resilience (see Figure 1).

**Figure 1.**
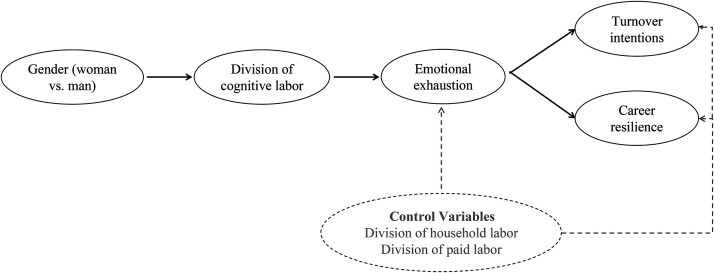
The Proposed Model in Which a Greater Proportion of Cognitive Labor within One’s Household and Emotional Exhaustion Serially Mediate the Negative Effect of Gender (Woman vs. Man) on Work-Related Outcomes (i.e., Turnover Intentions and Career Resilience).

We tested our hypotheses using a sample of employees from the United States and Canada with data collected toward the beginning of the pandemic (i.e., April–May 2020). We assessed participants’ experiences (i.e., division of cognitive labor, emotional exhaustion, turnover intentions, and career resilience) each week for 7 weeks. Although such a design captures both between- and within-person variation in these constructs, our primary focus was on between-person variance because gender is conceptualized as a between-person variable. Thus, we examined whether there were stable gender differences in cognitive labor aggregated across this period of time and the consequences of these differences. Moreover, in addition to testing our hypotheses, we explored the unique experiences of participants with and without children. Namely, given salient issues with childcare during the pandemic, which women were disproportionately responsible for (e.g., Calcaro et al., 2021), we examined the potentially unique roles of cognitive labor and childcare for women with children, and whether cognitive labor affected the work-related outcomes of women with and without children to the same degree.

Our research contributes to the literature on the gendered division of labor, which has only recently examined the mental work involved in managing a household (Daminger, 2019). Importantly, existing research on the topic has mainly examined the effects of cognitive labor on outcomes such as wellbeing and satisfaction (e.g., Reich-Stiebert et al., 2023). By focusing on turnover intentions and career resilience, we build on this past work by examining whether the division of cognitive labor, as well as the division of unpaid labor in general (Shockley et al., 2025), can spill over into the work domain. Further, our research stands to contribute to the extant literature by examining emotional exhaustion as an important underlying mechanism. In doing so, we answer calls in the literature to show *how* division of labor can impact work attitudes and intentions (Shockley & Shen, 2016), which can provide insight into psychological factors underlying gender differences in labor market outcomes (e.g., employment status and income). Overall, our research sheds light on this understudied, yet taxing and consequential, form of labor.

## Cognitive Labor

Past research on the division of unpaid labor within households has largely focused on physical labor, such as housework or childcare. In contrast, less visible forms of labor, such as mental or cognitive work, have often been overlooked (Eichler & Albanese, 2007) or have been lumped together with physical tasks (e.g., Hochschild, 1989), obscuring the pervasiveness of cognitive labor and its potentially unique impact on doers (e.g., Ciciolla & Luthar, 2019). Yet, cognitive labor is distinct from physical tasks and its primarily mental nature means it can be invisible yet also ubiquitous (Daminger, 2019). Cognitive labor underpins numerous other tasks and spans a number of domains (e.g., scheduling and finances). In addition, cognitive labor is likely particularly taxing because it involves open-ended tasks and leads to multitasking and time fragmentation (Ciciolla & Luthar, 2019; DeGroot & Vik, 2020).

Daminger's (2019) work was seminal in distinguishing cognitive labor from other forms of unpaid labor. Using qualitative data (i.e., interviews) with members of 35 heterosexual couples, Daminger developed a theoretical account of cognitive labor's underpinnings and established it as a gendered phenomenon. Namely, cognitive labor is made up of four distinct characteristics: anticipation (i.e., “recognition of an upcoming need, potential problem, or opportunity”), identification (i.e., “determining options for meeting the need”), decision making (i.e., “choosing among previously identified options”), and monitoring (i.e., “following up to ensure the decision is carried out and satisfactorily addresses the anticipated need”; Daminger, 2019, pp. 618–619). Daminger (2019) also identified the diffuseness, lack of time-boundedness, and invisibility of the components. Importantly, cognitive labor is regarded as a gendered phenomenon wherein women are more likely than men in heterosexual couples to engage in cognitive labor, particularly the anticipation and monitoring dimensions, which is high in invisibility and low in power (Daminger, 2019). By contrast, identification and decision making, which are low in invisibility as well as medium and high in power, respectively, are more likely to be shared between women and men.

Drawing on the literature on gender roles, we argue that women are responsible for a disproportionate amount of cognitive labor within their households, and consequently, experience more negative work outcomes than men. The psychological perspective on gender focuses on gender roles and suggests that they can operate as social norms that people strive to and are expected to uphold (Eagly, 1987; Prentice & Carranza, 2002). For example, men are expected to be agentic (e.g., aggressive and independent), whereas women are expected to be communal (e.g., supportive and helpful; Rudman et al., 2012). Generally, agency is self-oriented whereas communality is other-oriented and reflects concern for others’ wellbeing.

The sociological perspective on gender suggests that gender not only operates as a social norm, but as a social institution (Acker, 1992); it is not just something that outlines what people “are,” but something that they “do” (West & Zimmerman, 1987). Thus, beliefs about gender provide a blueprint for how people view themselves relative to others and how they should engage in everyday activities and interactions with others (Ridgeway & Correll, 2004). This can include deciding on how household tasks will be divided, as household tasks carry gendered connotations, which can act as a gender display and is a way to “do” gender (Ridgeway, 2011). Further, people are generally raised as gendered members of their household and acquire skills and experience that are related to their gender (Ridgeway, 2011). Thus, in heterosexual couples, the division of household tasks may also be based on the unique skills and experiences that women and men tend to already possess and bring to their shared households.

Drawing on these perspectives, we theorize that compared to men, women are apt to be disproportionately responsible for cognitive labor in their partnerships because it involves engaging in communal activities that ease the burden on other family members (e.g., anticipating issues, planning workarounds, and considering safety) and generally involves being caring and supportive. These behaviors are consistent with women's gender roles and may be a way in which women “do” gender. Further, through their experiences of being raised as gendered members of their households, cognitive labor may be something that women generally believe they already have more skills and experience with. As a result, couples may feel that women are better suited to take on the additional cognitive labor that may occur during uncertain and unprecedented times, such as those brought on by the pandemic.

Consistent with these arguments, prior research has found that women, relative to men, generally tend to do the majority of cognitive labor (Reich-Stiebert et al., 2023). Women are also more likely than men to take responsibility for household wellbeing (Doucet, 2006). Further, when heterosexual couples rationalize traditional divisions of household labor, they often do so by drawing on gender-specific skills and characteristics of each member (Daminger, 2020). Given that cognitive labor is an extension of the unpaid work that women tend to take responsibility for to support those within their households and is one way in which women can “do” gender, we argue that women are more likely to engage in a disproportionate amount of cognitive labor relative to their men partners.

*Hypothesis 1:* Women (vs. men) are responsible for a higher proportion of cognitive labor within their household.

## Consequences of Cognitive Labor

We further anticipate that women's greater engagement in cognitive labor is resource draining (Hobfoll, 1988). Specifically, taking on a greater proportion of cognitive labor is positively related to feelings of emotional exhaustion, which involves the depletion of psychological and emotional resources (Maslach & Jackson, 1981). Emotional exhaustion is a key underlying mechanism to consider given the primarily psychological and taxing nature of cognitive labor (Daminger, 2019; DeGroot & Vik, 2020).

Conservation of resources theory specifies that people are motivated to gain, retain, and protect valued resources because they help individuals manage stress and overcome challenges (Hobfoll, 1988). Thus, people are threatened by the actual or potential loss of resources because failing to protect or replenish them reduces coping capacity (e.g., Freedy & Hobfoll, 1994). We argue that cognitive labor is draining, above and beyond individuals’ other demands (i.e., paid labor and housework). This is because the unique nature of cognitive labor—being ongoing, open-ended, and invisible (Ciciolla & Luthar, 2019)—is particularly conducive to resource loss, as people may struggle to disengage from or offload mental responsibilities.

Due to their greater engagement in this taxing form of labor, working women may be particularly emotionally exhausted. Emotional exhaustion is a key component of burnout and is accompanied by psychological and emotional drain and physical fatigue (Wright & Cropanzano, 1998). Past theorizing indicates that low personal resources are related to emotional exhaustion (Trougakos & Hideg, 2009). According to conservation of resources theory, people take steps to protect existing resources when they are in a state of emotional exhaustion with already limited resources (Hobfoll, 1988). As such, to protect or regain resources, women could consider reducing time and effort in the work domain, such as by leaving their organization. Furthermore, traditional gender roles already emphasize that women's priorities should be in the family (vs. work) domain (Hochschild, 1989) and research has shown that, in general, women are more likely than men to quit their jobs (Hom et al., 2008) and sacrifice their career for their husband's (Ullrich et al., 2015). Thus, disengaging from their careers may be viewed as a more plausible option for some women and necessary to alleviate exhaustion.

We clarify that the option for women to leave their jobs and disengage from their careers may be a function of other social categories (e.g., race, social class, etc.) that intersect to determine the level of privilege or oppression experienced in the workplace. Namely, although White women may experience disadvantages compared to White men, they often experience greater privilege in the workplace than women of color (Holvino, 2010). For example, women of color are more likely to experience discrimination in hiring or promotion as well as barriers to socialization within organizations, and they often have more limited informal networks compared to White women (Amis et al., 2020). Thus, leaving one's job may prove to be more difficult and have stronger negative career implications for women of color. Social class, which is often conflated with race (Collins, 2000), may also impact women's turnover intentions. For example, being in a lower socioeconomic bracket may prevent women from leaving their jobs because their loss of income may be less likely to be offset by their partner's income and they may be less likely to have savings to help sustain their household.

We further argue that resource drain can also diminish coping capacity (Freedy & Hobfoll, 1994). In addition to strengthening turnover intentions, emotional exhaustion can also negatively impact career resilience. Those experiencing resource loss are likely to have a lowered capacity to cope with and be resilient to uncontrollable or discouraging changes in the workplace (Day & Allen, 2004). Besides experiencing organizational changes, women may have also felt pressure to manage the disruptions from and lessen the burden of the pandemic on their household as a way to “do” gender (Ridgeway, 2011). In addition, women tend to have broader and more varied life goals than men, in part because they may seek to avoid the conflict and risk they associate with focusing primarily on career advancement (Gino et al., 2015). The increased conflict between these sets of demands during the pandemic may have made this trade-off especially salient for women, undermining their ability or desire to cope effectively in the work domain. Furthermore, given that the pandemic necessitated changes in many organizations, lowered career resilience may have been particularly harmful. On the basis of this body of work, we hypothesized the following serial mediation model:

*Hypothesis 2:* Responsibility for a greater proportion of cognitive labor within one's household and emotional exhaustion serially mediate the negative effect of gender (woman vs. man) on work outcomes.

## The Role of Childcare

Another important source of unpaid labor during the pandemic was childcare due to the closure of schools and daycare facilities (Calcaro et al., 2021). This means that, in addition to disruptions to people's everyday lives and a shift in work arrangements for many, dual-earner couples with children had to juggle their work responsibilities with their childcare responsibilities, including guiding their children through remote learning (Garbe et al., 2020). Indeed, research has shown that women maintained greater responsibility for this form of labor during this time (Calcaro et al., 2021; Giurge et al., 2021). This had important implications for women's work outcomes. Namely, mothers who continued their employment during the pandemic were more likely to reduce their work time compared to fathers (Collins et al., 2021) and their employment recovery has been slower (Fuller & Qian, 2021).

This suggests that work outcomes of women with children may have been particularly affected by childcare responsibilities compared to women without children. This raises the question of whether an unequal division of cognitive labor affects women with and without children to the same degree, and whether it is a stronger predictor of work outcomes for women without children. Given that we did not originally set out to examine the impacts of childcare on parents’ work outcomes, and our sample consists of both parents and nonparents, we did not have a specific hypothesis regarding the potential or relative impact of childcare versus cognitive labor. Rather, we examined in an exploratory fashion whether the division of childcare among participants who are parents may uniquely impact mothers’ strain and work outcomes.

## Method

### Participants and Procedure

Participants were 263 individuals based in the United States (86%, *n* = 227) or Canada (14%, *n* *=* 36) who self-identified as men (48%, *n* = 125) or women (52%, *n* *=* 138) and completed our initial baseline survey and seven weekly surveys. They were employed and reported being in a heterosexual relationship with an employed partner. The majority of our participants were married (74%, *n* = 194) and approximately half (47%, *n* = 123) had children under the age of 18 who lived with them. Participants self-identified as White (80%, *n* = 210), Asian (7%, *n* = 19), Latino (5%, *n* = 14), Black (2%, *n* = 6), or multiracial/other (5%, *n* = 14). They were highly educated (87% had completed or were completing a four-year college or university degree or other postsecondary education, *n* = 230) and worked in various industries (e.g., finance, manufacturing, legal, and healthcare). On average, participants were 37 years old (*SD* = 9.38) and had been employed at their current organization for 6.42 years (*SD* = 5.99).

The study reported in this article was reviewed and approved by the Institutional Review Board prior to data collection (protocol #6505: “Understanding couples’ work and home experiences”). The current study was not part of a larger project. A total of 396 participants were recruited from Prolific, an online platform that assists researchers in recruiting participants (Peer et al., 2017), for a seven-wave study near the beginning of the COVID-19 pandemic, from April to May 2020. Participants were paid £2.00 for an initial baseline survey (approximately $2.50 US dollars and $3.50 Canadian dollars) and £1.25 (approximately $1.55 US dollars and $2.20 Canadian dollars) for each of the subsequent seven weekly surveys. Of the 396 participants who completed the initial baseline survey, 18 participants were not invited to complete the subsequent weekly surveys for various reasons (e.g., asked for their data not to be used, did not respond to questions of interest, provided inconsistent responses, etc.; one participant completed the survey twice with different responses). Overall, 378 participants were invited to participate in each of the weekly surveys regardless of whether they participated in the previous wave. Following best practices for online survey research (Cheung et al., 2017; Meade & Craig, 2012), we excluded 12 participants who did not correctly answer 2 of 3 attention checks (e.g., “respond to this item with strongly disagree”) or who asked for their data not to be used. We also excluded 12 participants who stated that they had been laid off in the baseline survey and 60 participants who stated that their partner was not employed in the baseline survey. Given that we were interested in examining participants who were in a heterosexual relationship, we excluded (a) 26 participants who reported that they and their partner identified as the same gender and (b) three participants who did not identify as a man or woman (two participants identified as nonbinary and one participant preferred not to answer the question; participants were given the option of choosing one of the following four response options for this question: *male*, *female*, *other* with an open-response option, and *prefer not to answer*). Finally, two participants did not complete any of the seven weekly surveys.

The final sample size was 263, but the sample size for the seven weekly surveys varied from 222 to 258 participants, as not all participants completed each weekly survey. We estimated that the effect size was likely small to medium. According to Cohen (1992), with α = .05 and for a medium effect size, a sample size of 64 is needed for sufficient statistical power to detect mean differences (β = .80), whereas for a small effect size, a sample size of 393 is necessary for sufficient statistical power. As the average of these two estimates is 228.5, we aimed to initially recruit approximately 280 individuals (to account for attrition and missing data).

In the seven weekly surveys, participants reported the division of cognitive labor, household labor, paid labor, and childcare as well as emotional exhaustion, turnover intentions, and career resilience over the past week. Although we were primarily interested in between-person differences due to our focus on gender, there remain key advantages to collecting repeated and aggregated measurements. Specifically, by asking participants to recall division of labor close in time to when it occurred, we could minimize retrospective biases (Gabriel et al., 2019). Moreover, doing so over the course of several weeks ensured that we had stable, person-level estimates of division of labor during this phase of the pandemic (i.e., results will be less affected by an anomalous week). Note that we examined whether there was cross-level moderation of gender on within-person associations in our model (e.g., was the within-person association between division of cognitive labor and emotional exhaustion different for women vs. men) but found no evidence of such effects.

We are unaware of any instances where two partners from the same household participated in our study and thus were not able to account for this in our analyses. However, on Prolific, there is an option to report that you are willing to participate in studies with your romantic partner who also has a Prolific account. Currently, Prolific screeners show that this reflects a relatively small proportion of Prolific participants (i.e., 7%). In addition, for many of these couples, both parties are not working in paid employment and therefore would not qualify for our study. Thus, we anticipate that if this did occur, it would be a rare occurrence and unlikely to bias our results.

### Division of Cognitive Labor

Division of cognitive labor was assessed with four items (average α = .92) that drew upon Daminger's (2019) qualitative work and identified four components: anticipate (i.e., “anticipating and recognizing upcoming needs, problems, or opportunities”), identify (i.e., “determining options for fulfilling needs”), decide (i.e., “choosing among previously identified options”), and monitor (i.e., “ensuring decision was executed and need sufficiently addressed”). Higher mean scores indicate engaging in a higher proportion of cognitive labor compared to one's partner.

As this was a new measure, we sought additional validation evidence by collecting data from a separate sample of 275 participants (50% women; 73% White; *M_age_* = 42.28, *SD_age_* = 11.13) recruited from Prolific who were living in the United States or Canada, working full-time, identified as heterosexual, and currently living with a partner. Participants reported on their typical division of cognitive labor (α = .90) over the past week, and an exploratory factor analysis indicated that all four items strongly loaded onto one factor. Demonstrating convergent validity, division of cognitive labor was moderately positively correlated (*r* = .41, *p* < .001) with division of prospective memory (i.e., memory for future actions and intentions), which has been argued to undergird and support cognitive labor (Harrington & Reese-Malancon, 2022). Demonstrating criterion-related validity, division of cognitive labor was negatively correlated with relationship satisfaction (*r *= −.18, *p *= .003), in line with prior research (Reich-Stiebert et al., 2023). Furthermore, we also corroborate associations we observed from the study reported in-text, such as associations between division of cognitive labor and division of other types of unpaid labor (i.e., household and childcare) as well as with emotional exhaustion. Overall, the results of this study provided evidence of convergent, discriminant, and criterion-related validity. For more details on this validation effort, please see the online supplemental materials.

### Emotional Exhaustion

Emotional exhaustion was measured using Maslach and Jackson's (1986) nine-item measure (e.g., “I have felt emotionally drained,” “I have felt used up at the end of the day,” “I have felt like I’m at the end of my rope;” 1 = *not at all* to 7 = *a great extent*). It is a subscale of the Maslach Burnout Inventory and reflects one factor. Higher mean scores indicate higher experience of emotional exhaustion over the past week. In prior research, internal consistency was reported as .90 and test–retest reliability was reported as .82 and .54 over a period of 2 to 4 weeks and 1 year, respectively (Maslach & Jackson, 1986). Average alpha for the current sample was .96. Validity was supported by its (a) positive correlations with personal experiences (i.e., reactions to clients, appearance of physical fatigue, arriving home from work upset and angry, tense or anxious, and complaining about work problems), feedback from the job, and personal outcomes (e.g., growth satisfaction, knowledge of job performance, absenteeism), as well as (b) low correlation with job satisfaction and no correlation with social desirability (Maslach & Jackson, 1981).

### Turnover Intentions

Turnover intentions were measured with Steffens et al.'s (2018) three-item measure (i.e., “I have thought about quitting my job,” “I would like to work for another organization in the short term,” and “I would like to leave this organization;” 1 = *not at all* to 7 = *very much*), which was adapted from Konovsky and Cropanzano (1991) and reflects one factor. Higher mean scores indicate greater intentions to leave one's job. Internal consistency was previously reported as .84 (Konovsky & Cropanzano, 1991). Average alpha for the current sample was .95. We used this scale because it has high face validity and it has been commonly used in research examining work attitudes (e.g., Randall et al., 1999; Shen et al., 2024).

### Career Resilience

Career resilience was measured with Day and Allen's (2004) seven-item measure (e.g., “I was able to adapt to changing circumstances,” “I welcomed job and organizational changes,” “I adequately handled work problems that came my way;” average α = .87; 1 = *not at all* to 7 = *very much*). Career resilience is a subscale of career motivation. Higher mean scores indicate stronger perceptions of being able to adapt to changing circumstances. Day and Allen's (2004) career motivation scale was adapted from two existing scales, both of which are made up of the same three subscales (i.e., career resilience, career insight, and career identity). Internal consistency was previously reported as .84 (Day & Allen, 2004). Average alpha for the current sample was .87. Validity of the two existing scales incorporated by Day and Allen (2004) has been previously established (Grzeda & Prince, 1997). Namely, validity for career resilience was supported by (a) positive correlations with creativity, autonomy, persistence, and perseverance and (b) showing that the career resilience measurement variable loaded on the predicted latent variable (Grzeda & Prince, 1997).

### Control Variables

To examine the unique role of division of cognitive labor beyond other forms of labor, we included the division of household and paid labor as control variables in our model. *Division of household labor* was measured with four items (i.e., “shopping for groceries,” “cooking meals,” “doing the dishes,” and “cleaning the house;” Mannino & Deutsch, 2007). This measure was developed by Mannino and Deutsch (2007) along with division of childcare, which is used as an exploratory control variable in this study (see below). Although the two dimensions reflected one factor, Mannino and Deutsch (2007) differentiated housework from childcare tasks on a conceptual basis. We adapted the original response scale to maintain consistency with the response scale used for division of cognitive labor. Higher mean scores indicate engaging in a higher proportion of household labor compared to one's partner. Internal consistency of a similar measure has been reported as .69 to .73 (Lozano & Garcia-Roman, 2022). Average alpha for the current sample was .63. This scale was used due to its high face validity and measures with similar items have been used in past research (e.g., Lozano & Garcia-Roman, 2022).

Two items assessed *division of paid labor* over the past week: one item on household income (1 = *my partner brought in nearly all of our household income* to 7 = *I brought in nearly all of our household income*) and the other on work hours (1 = *my partner spent much more time working* to 7 = *I spent much more time working*). This measure was developed for the purpose of this study. Higher mean scores indicate engaging in a higher proportion of paid labor compared to one's partner. Average alpha for the current sample was .80.

### Exploratory Measures

To examine in an exploratory fashion whether the division of childcare among participants who are parents uniquely impacts mothers’ strain and work outcomes, we assessed *division of childcare* using eight items for those who were parents (e.g., “helping your children get dressed in the morning,” “putting your children to bed,” “playing with your children,” “give your children a bath,” “making decisions about your children's upbringing,” “reading to your children,” “arranging for childcare,” and “responding to your children's requests and ongoing needs;” Mannino & Deutsch, 2007). Higher mean scores indicate engaging in a higher proportion of childcare compared to one's partner. Average alpha for the current sample was .87.

### Analytic Plan

First, we conducted a multilevel confirmatory factor analysis in Mplus, Version 8.1 (Muthén & Muthén, 2017) with six factors (i.e., division of cognitive labor, household labor, paid labor, emotional exhaustion, career resilience, and turnover intentions) at the week and person levels to establish model fit. The model demonstrated an adequate fit to the data, *χ*^2^ = 2085.98, *df* = 724, comparative fit index (CFI) = .90, Tucker-Lewis index (TLI) = .89, root mean square error of approximation (RMSEA) = .03, standardized root mean square residual (SRMR) (within) = .04, and SRMR (between) = .06. Our hypothesized model fit the data significantly better than a model with one factor at each level, Satorra-Bentler Δ*χ*^2^ = 2508.32, *df* *=* 30, *p* < .001 (*χ*^2^ = 8580.33, *df* = 754, CFI = .41, TLI = .37, RMSEA = .08, SRMR [within] = .11, SRMR [between] = .19) and a three-factor model with the three labor variables loaded onto one factor, emotional exhaustion loaded onto one factor, and the two outcome variables loaded onto one factor, Satorra-Bentler Δ*χ*^2^ = 1298.89, *df* = 24, *p* < .001 (*χ*^2^ = 4800.90, *df* = 748, CFI = .70, TLI = .67, RMSEA = .06, SRMR [within] = .07, SRMR [between] = .12).

Given the hierarchical structure of our data, we tested our hypotheses using multilevel structural equation modeling (Preacher et al., 2010) in Mplus. This analytical approach avoids conflating estimates of within- and between-persons indirect effects. In other words, the model accounts for the reality that individual participants can experience varying divisions of cognitive labor, emotional exhaustion, and work-related outcomes week-over-week. Although our hypotheses center on the between-person level of analysis, we needed to accurately model and partition the variance in our data to ascertain whether our hypotheses were supported. We used Bayesian estimation procedures, which allow for testing of multilevel mediation that does not assume or require a normal distribution of indirect effects (Yuan & MacKinnon, 2009).

The within-person (level 1) part of the model included weekly division of cognitive labor as a predictor of weekly emotional exhaustion and work-related outcomes. The between-person (level 2) part of the model specified gender as a person-level predictor of the *average* division of cognitive labor, emotional exhaustion, and work outcomes. We controlled for effects of division of household and paid labor on emotional exhaustion and work outcomes. Thus, our overall model assessed (a) whether gender predicts a person's average division of cognitive labor as reported across 7 weeks and (b) if this then predicts average emotional exhaustion and work outcomes beyond the effects of average division of household and paid labor.

## Results

Means, standard deviations, and correlations among variables aggregated across the seven weekly surveys are reported in Table 1. All variables varied both within- and between-persons, indicating multilevel analysis is appropriate. However, the majority of the variance across study variables was at the between-person level of analysis (ICCs ranging from .72–.85).

**Table 1.** Means, Standard Deviations, and Correlations Among All Participants.

Variable	*M*	*SD*	1	2	3	4	5	6	7	8
1. Gender	0.52	0.50								
2. Parental status	0.47	0.50	−.16**							
3. Household labor	4.46	1.21	.42**	−.02		−.13**	.09**	.10**	−.01	.00
4. Paid labor	4.11	1.50	−.38**	.02	−.38**		.04	.07**	.01	.10**
5. Cognitive labor	4.42	0.82	.27*	.04	.34**	−.08		.04	−.01	.01
6. Emotional exhaustion	2.87	1.51	.18*	.03	.13*	−.02	.22**		.13**	−.07**
7. Turnover intentions	2.56	1.82	.02	−.01	.05	−.06	.20**	.35**		−.02
8. Career resilience	5.00	1.07	−.13*	−.01	−.15*	.20**	−.02	−.34**	−.26**	

*Note*. Level 1 *n* = 1632–1635; Level 2 *n* = 263. Between-person correlations are reported below the diagonal and within-person correlations are reported above the diagonal. Gender is coded as 0 = man, 1 = woman. Parental status is coded as 0 = no children or no children under the age of 18 living with them, 1 = child(ren) under the age of 18 living with them. All three forms of labor refer to division of labor, such that higher scores mean that one was engaging in a higher proportion of this form of labor within the household relative to one's partner.

*p < .05. **p < .01.

### Preliminary Analyses

We conducted preliminary analyses to first understand different forms of labor during the study period, which spanned 2 months near the beginning of the COVID-19 pandemic. In the initial baseline survey, participants were asked whether they perceived that household labor and paid labor (within the household) had changed due to the COVID-19 pandemic (1 = *decreased* to 7 = *increased*). One-sample *t*-tests comparing each mean to the midpoint of the scale (4 = *stayed about the same*) indicated that household labor had generally increased (*M* = 4.75, *SD* = .96), *t*(262) = 12.63, *p* < .001, and paid labor had generally decreased (*M* = 2.95, *SD* = 1.43), *t*(262) = −11.92, *p* < .001. We also asked participants whether they perceived that cognitive labor had changed due to the COVID-19 pandemic with one question (i.e., “need for planning and decision-making regarding the household”) and a one-sample *t*-test indicated that cognitive labor had generally increased (*M* = 4.86, *SD* = .97), *t*(262) = 14.30, *p* < .001.

Further, independent samples *t*-tests indicated that there were no differences between women (household labor: *M* = 4.78, *SD* = .97; paid labor: *M* = 2.91, *SD* = 1.50; cognitive labor: *M* = 4.96, *SD* = 1.01) and men (household labor: *M* = 4.71, *SD* = .95; paid labor: *M* = 3.00, *SD* = 1.34; cognitive labor: *M* = 4.74, *SD* = .91) in whether they perceived changes in household labor, *t*(261) = 0.54, *p* = .593, paid labor, *t*(261) = −0.53, *p* = .594, or cognitive labor, *t*(261) = 1.91, *p* = .057, due to the COVID-19 pandemic. As such, whereas on average participants perceived that household, paid, and cognitive labor had all increased due to the COVID-19 pandemic, gender did not affect (i.e., strengthen or weaken) perceptions of such changes.

We were also interested in whether the *division* of labor was changing *during* our study (i.e., whether there were trends in participants’ perceptions that they were taking on consistently more or less labor compared to their partner). This required us to first test for measurement invariance to ensure that measures were equivalent across time (Ployhart & Vandenberg, 2010; for more details, please see the online supplemental materials). Results supported measurement equivalence for paid labor and cognitive labor (but not household labor or childcare). Thus, we only examined latent growth models (in Mplus) for division of paid labor and cognitive labor.

There was no consistent change in the division of paid (*γ* = .00, *SE* = .01, *p* = .859) or cognitive labor (*γ* = .00, *SE* = .01, *p* *=* .841) across all participants. Subgroup latent growth models also showed no consistent change for women or men in division of paid (women: *γ* = −.01, *SE* = .02, *p* = .792; men: *γ* = .00, *SE* = .01, *p* = .884) or cognitive labor (women: *γ* = .00, *SE* = .01, *p* = .883; men: *γ* = −.01, *SE* = .01, *p* = .523).

Overall, these preliminary analyses suggest that participants perceived an overall increase in household and cognitive labor due to the pandemic and a decrease in paid labor. However, there was no consistent change in the perceived *division* of labor between participants and their partners over the subsequent 7 weeks of the study. In other words, individual participants reported fluctuations across the study period, but on average across all participants, there was no consistent increase or decrease in how that labor was perceived to be divided within households. As there were no significant trends, this provides further support that focusing on the *average* division of cognitive labor across this period of time was an appropriate choice for this sample.

### Tests of Hypotheses

Results of between-persons analyses are reported in Table 2. Supporting hypothesis 1, women (vs. men) reported engaging in a higher proportion of cognitive labor within their household (*b* = .46, *SD* = .10, *p* < .001). Moreover, being responsible for a higher proportion of cognitive labor within one's household was positively associated with emotional exhaustion (*b* = .42, *SD* = .12, *p* < .001), beyond the effects of the proportion of household and paid labor in which participants engaged. In turn, emotional exhaustion was positively related to turnover intentions (*b* = .42, *SD* = .08, *p* < .001) and negatively related to career resilience (*b* = −.26, *SD* = .04, *p* < .001). Note that in Bayesian analysis, *SD* refers to the standard deviation of the posterior distribution and is an estimate of uncertainty comparable to the standard error in frequentist analysis.

**Table 2.** Between-Person Analyses of Relations.

	Mediator variables				Dependent variables			
	Cognitive labor		Emotional exhaustion		Turnover intentions		Career resilience	
Gender	.46**	(.10)	.36	(.21)	–.34	(.23)	–.02	(.14)
Cognitive labor			.42**	(.12)	.30*	(.15)	.17**	(.09)
Emotional exhaustion					.42**	(.08)	–.26*	(.04)
Household labor			.02	(.09)	–.06	(.11)	–.07	(.06)
Paid labor			.05	(.07)	–.10	(.08)	.12*	(.05)
*R* ^2^	.08*		.09*		.16**		.17**	

*Note*. Values are unstandardized estimates with standard errors in parentheses. Gender is coded as 0 = man, 1 = woman.

*p < .05. **p < .01.

**Table 3.** Means, Standard Deviations, and Correlations Among Participants Who Have a Child Under the Age of 18 Living With Them.

Variable	*M*	*SD*	1	2	3	4	5	6	7	8
1. Gender	0.44	0.50								
2. Household labor	4.44	1.23	.49**		−.19**	.09*	.17**	.07	−.05	.01
3. Paid labor	4.14	1.58	−.46**	−.43**		.06	−.08*	.06	.05	.12**
4. Cognitive labor	4.46	0.82	.30**	.46**	−.16		.08*	.04	.00	−.04
5. Childcare	4.32	1.00	.67**	.60**	−.60**	.45**		−.02	−.03	.04
6. Emotional exhaustion	2.92	1.65	.15	.20*	−.19*	.29**	.32**		.11**	−.03
7. Turnover intentions	2.54	1.80	−.09	.10	−.08	.18*	.11	.31**		.03
8. Career resilience	4.99	1.12	−.19*	−.19*	.31**	−.05	−.23*	−.52**	−.28**	

*Note*. Level 1 *n* = 760–763; level 2 *n* = 123. Between-person correlations are reported below the diagonal and within-person correlations are reported above the diagonal. Gender is coded as 0 = man, 1 = woman. All three forms of labor refer to division of labor, such that higher scores mean that one was engaging in a higher proportion of this form of labor within the household relative to one's partner.

*p < .05, **p < .01.

Further, supporting our full model and hypothesis 2, tests of the indirect effects (10,000 iterations) revealed that the indirect effect of gender on turnover intentions (indirect effect = .08, *SD* = .03, 95% CI [.021, .147]) and career resilience (indirect effect = −.05, *SD* *=* .02, 95% CI [−.091, −.014]) mediated serially through (a) division of cognitive labor and (b) emotional exhaustion was significant. Thus, women's (vs. men's) greater responsibility for cognitive labor was uniquely draining, beyond the effects of division of paid labor and housework.

### Exploratory Analyses

We also explored whether the results reported above held when also controlling for division of childcare among participants who had a child under the age of 18 living with them. Means, standard deviations, and correlations among participants who have a child under the age of 18 living with them are reported in Table 3. Generally, a one-sample *t*-test comparing each mean to the midpoint of the scale (4 = *stayed about the same*) indicated that participants reported that childcare had increased due to the pandemic (*M* = 5.07, *SD* = 1.76), *t*(122) = 6.78, *p* < .001. Further, an independent samples *t*-test indicated that women (*M* = 4.98, *SD* = 1.96) and men (*M* = 5.14, *SD* = 1.59) did not significantly differ in their perceptions of changes in childcare due to the pandemic, *t*(121) = 0.51, *p* = .611.

Among mothers (vs. fathers), being responsible for a higher proportion of cognitive labor in the household during the course of the study was not uniquely associated with emotional exhaustion (*b* = .40, *SD* = .20, *p* = .052). This appeared to be because unequal division of childcare was strongly related to emotional exhaustion. Namely, mothers (vs. fathers) reported engaging in a higher proportion of childcare within their household (*b* = 1.35, *SD* = .14, *p* < .001). In turn, being responsible for a higher proportion of childcare was positively associated with emotional exhaustion (*b* = .55, *SD* = .25, *p* = .030), beyond the effects of proportion of cognitive, household, and paid labor one engaged in relative to one's partner. Tests of the indirect effects (10,000 iterations) further revealed that the indirect effect of gender on career resilience (indirect effect = −26, *SD* *=* .14, 95% CI [−.542, −.009]) mediated serially through (a) division of childcare and (b) emotional exhaustion was significant, but the effect on turnover intentions was not significant (indirect effect = .20, *SD* = .14, 95% CI [−.009, .497]).

These exploratory results indicate that mothers (vs. fathers) engaged in a higher proportion of childcare, which was related to higher emotional exhaustion and, subsequently, lower career resilience. At the same time, the division of cognitive labor was not a significant predictor of emotional exhaustion for mothers (vs. fathers). Overall, our results show that engaging in a higher proportion of cognitive labor uniquely affected the work outcomes of women without children, whereas engaging in a higher proportion of childcare shaped the work outcomes of women with children.

### Supplemental Analyses

One concern that could be raised about our analytic approach is that our conceptual model is directional, implying a temporal sequencing that is not reflected in our analyses above. Therefore, we also conducted supplemental analyses based on time-separated measurements in which we estimated division of cognitive labor (as well as household, paid labor, and childcare) by averaging responses to weekly surveys 1, 2 and 3, emotional exhaustion by averaging responses to weekly surveys 4 and 5, and turnover intentions and career resilience by averaging responses to weekly surveys 6 and 7. However, a notable trade-off to this approach is that these analyses are based on less reliable between-person estimates of these constructs (i.e., aggregating across only two or three observations as opposed to seven). The pattern of results and conclusions were congruent with our hypotheses tests and exploratory analyses reported above (for more details, please see online Supplemental materials). Thus, when women were unduly responsible for cognitive labor in their households for a period of time, they subsequently experienced greater emotional exhaustion, which then contributed to greater turnover intentions and lower career resilience at a later time.

## Discussion

Our study shows that cognitive labor, an invisible but taxing form of unpaid labor involved in managing a household, can serve as a source of emotional exhaustion that harms women's work outcomes. In the context of the COVID-19 pandemic, we found that women (vs. men) engaged in a disproportionate amount of cognitive labor and experienced higher emotional exhaustion, higher turnover intentions, and lower career resilience as a result. Thus, unequal engagement in this form of unpaid labor may have spilled over into the work domain and may have led women to consider exiting the workforce or impacted their ability to cope with changes in their work environment, to the detriment of workplace gender equity. This greater responsibility for cognitive labor appears to undermine women's work outcomes above and beyond other more commonly studied types of unpaid labor that women (vs. men) are also disproportionately responsible for (i.e., household labor).

We further found that these findings are most relevant for partnered women without children. That is, our exploratory analyses showed that women's disproportionate responsibility for childcare was the main source of emotional exhaustion for mothers, despite mothers (vs. fathers) also taking on a larger share of cognitive labor within their households. As such, although both women with and without children experienced higher levels of emotional exhaustion and consequent undermined work outcomes, the primary driver of these outcomes differed. That is, division of cognitive labor was uniquely related to the work outcomes of women without children, whereas childcare was uniquely related to the work outcomes of women with children.

One reason why we may not have found an effect of cognitive labor for mothers could be the timing of our data collection, which took place during the early phases of the pandemic. It coincided with initial prolonged school and daycare closures and was an unprecedented situation for many families. As such, childcare responsibilities were especially overwhelming during this time and may have made this form of labor particularly salient. Alternatively, this may also have been due to the sample size as less than half of participants (47%, *n* = 123) had children under the age of 18 who lived with them. Given that the effect of division of cognitive labor for mothers (vs. fathers) was marginally significant, this may mean that we did not have sufficient power to detect an effect. Further, it is important to note that there is a cognitive element to engaging in childcare (e.g., responding to children's ongoing needs or arranging childcare). As such, there is also the possibility that, for mothers, cognitive labor is consumed by childcare, which may have particularly been the case during the pandemic. In contrast, cognitive labor may relate more generally to household management for women without children.

In addition, we note that emotional exhaustion contributed to both greater turnover intentions and undermined career resilience among women without children, but only negatively affected career resilience for women with children. This may reflect that mothers are likely to be “reluctant stayers.” In other words, despite exhaustion, they may have felt unable to leave their organization due to financial pressures to support their family and were coping in other ways (e.g., decreasing their work hours; Collins et al., 2021).

### Theoretical Implications

This article contributes to the current understanding of how division of unpaid labor within households affects work outcomes, both during the pandemic and beyond. In the context of the pandemic, we provide further evidence that women in heterosexual relationships continue to be disproportionately responsible for household labor, despite some men and women corroborating that men partners increased their engagement in unpaid labor during this time (Shafer et al., 2020). Namely, this modest change was insufficient to offset the increase in unpaid labor during the pandemic (i.e., cognitive labor and childcare), for which the women in our study reported undue responsibility. Thus, relative to their men partners, women appeared to be working a “third shift” that led to greater emotional exhaustion (after controlling for division of household and paid labor). This in turn had negative effects on women's work outcomes.

Our findings also provide key insights into the impact of gender roles in sustaining gender inequity within households. Scholars have pointed out that traditional gender roles are crucial in maintaining unequal division of labor within households, with women tending to be responsible for work within the home and men being responsible for work outside the home (e.g., Acker, 1992). We find that women also disproportionately shouldered an additional mental load within their households, despite the fact that many of the challenges associated with this pandemic (e.g., keeping up with changing information and guidelines) were novel and unprecedented. This lends support to the argument that although changes in household circumstances can be a catalyst for renegotiating the division of unpaid labor, they often fail to produce lasting change (Mannino & Deutsch, 2007). As our study shows, even remarkable shifts in the home and employment landscape may be unable to meaningfully shift entrenched divisions of labor within households.

Our article also contributes to the broader literature on the consequences of division of household labor. The limited past research on the career consequences of unequal division of unpaid labor has typically studied the impact of the “second shift” on job status or income (e.g., Lachance-Grzela & Bouchard, 2010). We build on this existing literature by shedding light on intentions and attitudes (i.e., turnover intentions, career resilience) that may help explain these labor-market outcomes. In addition to this, our work underscores the importance of examining the unique experiences of partnered women both with and without children as they are often grouped together in the literature. Namely, our findings suggest that disproportionate engagement in unpaid labor can differentially negatively impact women's wellbeing and workplace outcomes (i.e., childcare for women with children and cognitive labor for women without children), and they highlight the importance of taking into account these unique circumstances.

This also has implications for our understanding of turnover intentions. Prior research has shown that women are more likely than men to leave their organization (Hom et al., 2008). Our findings show that women without children, but not women with children, experienced greater turnover intentions. This contributes to knowledge of gender differences in turnover intentions by providing insight into the circumstances under which women may be more or less likely to leave their jobs. Further, this paper sheds much needed light on an understudied form of unpaid labor—cognitive labor—and begins to elucidate its work consequences, expanding upon the nascent work on this topic that has typically focused on relationship or wellbeing outcomes (e.g., Ciciolla & Luthar, 2019; Reich-Stiebert et al., 2023).

Importantly, we also uncovered emotional exhaustion as a psychological mechanism through which unequal division of labor can negatively impact work-related intentions and attitudes. Namely, our paper explored *how* the entrenchment and exacerbation of existing labor imbalances between men and women can set back progress toward gender equity. Specifically, our results suggest that taking on a disproportionate responsibility for cognitive labor or childcare is emotionally draining, which may have downstream consequences for women's work outcomes. More broadly, it can impact gender equity at work and in our labor force. Without some rebalancing of these heavy second and third shifts, women may be more likely to feel exhausted and attempt to cope by disengaging from paid employment (e.g., leaving their organizations) or be less resilient to the changes or obstacles that arise in their work and careers.

### Limitations and Future Research Directions

We recognize some limitations of our research. First, our study assessed *individual perceptions* of how labor was divided within participants’ households. Future research surveying both partners within a household would allow for additional insights as to what extent there is agreement regarding perceptions of division of labor. This may be especially important for cognitive labor because the relative invisibility of mental work may mean that partners are more likely to disagree about how this type of labor is distributed. Further, although we have developed and validated a measure of cognitive labor, one potential limitation is that some items were double-barreled, which goes against best practices for scale development (e.g., Cortina et al., 2020). Yet, some researchers have argued that the use of highly synonymous terms within an item connected by an “and,” which we believe applies in our current case, may be less problematic than using “and” for very distinct terms (Bartkus et al., 2015). Given these ambiguities, future work that continues to refine and validate this measure may be needed.

Second, our measures of work outcomes (i.e., turnover intentions and career resilience) captured intentions and attitudes rather than actual work behaviors. However, the theory of planned behavior and corresponding empirical research suggests that attitudes and intentions account for considerable variance in behaviors, with intentions serving as immediate predictors of behaviors (Ajzen & Fishbein, 1977). As such, attitudes and intentions are key predictors of behaviors. We would also like to note that although undermined career resilience is an attitude, it may have powerful adverse downstream effects on women's career trajectories and exacerbate gender inequity, as it may negatively affect women's work performance and lead to fewer workplace rewards, opportunities, and promotions compared to men.

Third, our sample is mostly White and highly educated, which is consistent with most convenience samples (e.g., Cheung et al., 2017) but is not fully representative of the dual-earner population in the United States and Canada. Thus, future research should take an intersectional lens to examine how cognitive labor is divided within a more racially or socioeconomically diverse range of households (e.g., whether non-White women experience cognitive labor differently) and the impact on work outcomes (e.g., whether partnered childless women who are lower in socioeconomic status and may not be able to rely on their partner's income would experience the same level of turnover intentions). Similarly, future research should consider different living situations (e.g., single women and men with or without children) in which people have no choice but to take on a high amount of cognitive labor within their household and do not have the financial support of a partner to lean on. Furthermore, based on evidence that traditional gender roles do not necessarily shape division of labor in the same way for same-sex couples (e.g., Goldberg et al., 2012), future research should also investigate how same-sex, transgender, or nonbinary couples manage cognitive labor demands.

Fourth, all of our measures were collected at the same time each week, which may increase concerns regarding common method bias. However, we attempted to minimize this in several ways (Podsakoff et al., 2003, 2012). For instance, we used different scale anchors across measures where possible and provided a second set of instructions between measures (a) focusing on division of labor and (b) our other mediator and outcome variables to create psychological separation. In addition, we attempted to reduce ambiguity by asking participants about their experiences over the past week. Finally, supplemental analyses reveal that our model and conclusions remain robust when tested with time-separated measurements.

Finally, some of our discourse on cognitive labor (e.g., theorizing and discussion of our findings) relates to the COVID-19 pandemic given that our data was collected at the beginning of the pandemic (April–May 2020) and may have been influenced by this context. However, given the nature of our data, we do not infer that our findings are a result of the pandemic itself, but rather clarify that our study was situated *during* the pandemic. Future research should examine the causal impact of the pandemic on women's and men's engagement in unpaid labor and the subsequent impact on their well-being and work outcomes. Further, although the results of our preliminary analyses suggest that various forms of unpaid labor, including cognitive labor and childcare, may have increased due to the pandemic, this does not necessarily mean that *associations* between variables changed as a result of the pandemic. Indeed, we note that the results of our validation study, which was conducted postpandemic (May 2023), provides support for the generalizability of some of our findings (i.e., a positive association between division of cognitive labor and emotional exhaustion). Nonetheless, future research should continue to examine the generalizability of our findings beyond the pandemic.

### Practical Implications

The first implication of our work is the necessity of bringing awareness to cognitive labor, an invisible form of labor that women tend to unequally engage in. Popular media or public discourse often discuss unequal division of household labor and childcare responsibilities but absent from this discourse is cognitive labor. As our work shows, this form of labor can negatively influence women—particularly the work outcomes of childless women. This is important as division of unpaid labor on childless women tend to be minimized and overlooked.

Our findings also have implications for organizations. Managers need to be cognizant that (a) their employees have various demands and (b) that women often have a greater burden of cognitive labor, which has negative work consequences. Our work also brings to light the need for managers to provide family supportive supervision (Hammer et al., 2009) to both fathers and mothers (or to employees with other caregiving responsibilities, e.g., eldercare). Extending similar supports to fathers may be crucial in helping them take proportionate responsibility for childcare, which is in turn often beneficial for their women partners and their careers (e.g., Hideg & Krstic, 2021; Hideg et al., 2023). Our work also highlights that support for employees with children is not enough and that women without children are also bearing responsibilities that can impact their wellbeing and work outcomes. To ensure an equitable and inclusive workplace, organizations need to support women and, more broadly, employees without children too.

Finally, our results have implications for policymakers. The COVID-19 pandemic illuminated and exacerbated the disparities in household and childcare labor. Indeed, there have been calls worldwide for policymakers to apply a gender lens in pandemic-related management and recovery efforts (e.g., Risse, 2021), and our findings reinforce this need. However, beyond the pandemic, policymakers must also recognize that women's unequal engagement in unpaid labor may impact their wellbeing and work outcomes, regardless of whether they have children or not. Thus, our work highlights the struggles of women without children and brings attention to the need for policymaking that is more inclusive of all women, not just women with children, to better promote gender equity.

## Conclusion

The unequal division of unpaid labor, which women often take disproportionate responsibility for, has been argued to be a major barrier to gender equity. Drawing on the literature on gender roles and conservation of resources theory, our findings support these contentions in that women's (vs. men's) disproportionate engagement in these emotionally taxing forms of unpaid labor—cognitive labor for women without children and childcare for mothers—appear to have led to higher emotional exhaustion and, in turn, seems to have harmed their work outcomes (i.e., turnover intentions, career resilience). Although concerning, recognizing the gendered impacts of unpaid labor is crucial for the promotion of workplace gender equity. This article is but one step in beginning to elucidate the consequences of unpaid labor on women's work outcomes; further research is needed to further clarify these effects.

## Supplemental Material

sj-docx-1-pwq-10.1177_03616843251330284 - Supplemental material for Taking on the Invisible Third Shift: The Unequal Division of Cognitive Labor and Women’s Work Outcomes Supplemental material, sj-docx-1-pwq-10.1177_03616843251330284 for Taking on the Invisible Third Shift: The Unequal Division of Cognitive Labor and Women’s Work Outcomes by Anja Krstić, Winny Shen, Christianne T. Varty, Janice Y. Lam and Ivona Hideg in Psychology of Women Quarterly
