# Supplementary material for: Taking on the Invisible Third Shift: The Unequal Division of Cognitive Labor and Women’s Work Outcomes
Source: Psychol Women Q. 2025 Apr 21;49(2):205–19. doi: 10.1177/03616843251330284 (PMC12058002; doi:10.1177/03616843251330284)
Supplement: sj-docx-1-pwq-10.1177_03616843251330284 - Supplemental material for Taking on the Invisible Third Shift: The Unequal Division of Cognitive Labor and Women’s Work Outcomes [file sj-docx-1-pwq-10.1177_03616843251330284.docx]

**Online Supplemental Materials**

**Taking on the Invisible Third Shift: The Unequal Division of Cognitive Labor and Women’s Work Outcomes**

**In-Text Study** **Measures**

**Division of cognitive labor (Daminger, 2019)**

In the past week, between you and your partner, what was the typical distribution among the following? (1 = *my partner did nearly all of this*, 4 = *we were sharing this equally*, 7 = *I did nearly all of this*)

1. Anticipating and recognizing upcoming needs, problems, or opportunities.
2. Determining options for fulfilling needs.
3. Choosing among previously identified options.
4. Ensuring decision was executed and need sufficiently addressed.

**Emotional exhaustion (Maslach & Jackson, 1986)**

Please rate the extent to which you have felt the following in the past week (1 = *not at all* to 7 = *to a great extent*):

1. I have felt emotionally drained.
2. I have felt used up at the end of the day.
3. I have felt fatigued when I get up in the morning and have to face another day.
4. Being around people all day has really been a strain on me.
5. I have felt burnt out.
6. I have felt frustrated.
7. I have felt I’m working too hard.
8. Working with people has put too much stress on me.
9. I have felt like I’m at the end of my rope.

**Turnover intentions (Steffens et al., 2017)**

Please rate the extent to which you have felt the following in the past week (1 = *not at all* to 7 = *very much*):

1. I have thought about quitting my job.
2. I would like to work for another organization in the short term.
3. I would like to leave this organization.

**Career resilience (Day & Allen, 2004)**

Thinking about your work from the past week, please rate the extent to which you agree with the following (1 = *not at all* to 7 = *very much*):

1. I was able to adapt to changing circumstances.
2. I was willing to take risks (outcomes with uncertain outcomes).
3. I welcomed job and organizational changes.
4. I adequately handled work problems that came my way.
5. I believed other people when they told me that I did a good job.
6. I designed better ways of doing my work.
7. I outlined ways of accomplishing jobs without waiting for my boss.

**Division of household labor (Mannino & Deutsch, 2007)**

In the past week, between you and your partner, what was the typical distribution of effort among the following? (1 = *my partner did nearly all of this*, 4 = *we were sharing this equally*, 7 = *I did nearly all of this*):

1. Shopping for groceries
2. Cooking meals
3. Doing the dishes
4. Cleaning the house

**Division of paid labor**

Over the past week, between you and your partner:

1. What was the distribution of household income? (1 = *my partner brought in nearly all of our household income*, 4 = *we contributed equally to the household income*, 7 = *I brought in nearly all of our household income*)
2. What was the distribution of work hours? (1 = *my partner spent much more time working*, 4 = *we worked equal hours*, 7 = *I spent much more time working*)

**Division of childcare (Mannino & Deutsch, 2007)**

In the past week, between you and your partner, what was the typical distribution of effort among the following? (1 = *my partner did nearly all of this*, 4 = *we were sharing this equally*, 7 = *I did nearly all of this*)

1. Helping your children get dressed in the morning
2. Putting your children to bed
3. Playing with your children
4. Give your children a bath
5. Making decisions about your children’s upbringing
6. Reading to your children
7. Arranging for childcare
8. Responding to your children’s requests and ongoing needs

**Division of Cognitive Labor Scale Validation Study**

The aim of this study was to validate the division of cognitive labor measure used in our study in-text. This measure was developed based on Daminger’s (2019) qualitative work. In this validation study, we first conducted an exploratory factor analysis to examine the factor structure of the measure and then examined evidence for convergent, discriminant, and criterion-related validity. Convergent validity represents the extent to which a scale relates to measures of similar constructs, whereas discriminant validity represents the extent to which it is not correlated or shows low correlations with dissimilar constructs (Hinkin, 1998). Criterion-related validity represents the relationship between the measure and variables which they theoretically predict (Hinkin, 1998). This study was preregistered on the Open Science Framework website (anonymized link: https://osf.io/v4bqw?view_only=0fc0d09f73924ece9fd5e8c7f70ce6de).

Specifically, to assess convergent validity, we tested whether division of cognitive labor is positively correlated with measures of similar, related constructs, such as division of household labor, division of childcare, and division of emotion work (i.e., maintaining others’ emotional well-being and providing emotional support; Erickson, 1993). We chose these variables because, similar to division of cognitive labor, they represent unpaid, family-oriented labor as well as caring for others. We also tested whether it is positively correlated with division of prospective memory, which refers to “memory for future actions” and includes making plans, providing reminders, and ensuring things are accomplished (Harrington & Reese-Melancon, 2022, p. 190). We chose it as, similar to cognitive labor, it is also form of invisible mental labor.

To assess discriminant validity, we examined whether division of cognitive labor is uncorrelated with dissimilar constructs, such as division of paid labor, social desirability (i.e., the tendency to present oneself in an overly positive way; Paulhus, 2002), and political skill (i.e., “the ability to effectively understand others at work, and to use such knowledge to influence others to act in ways that enhance one’s personal and/or organizational objectives;” Ahearn et al., 2004, p. 311; Ferris et al., 2005). Although both division of cognitive labor and paid labor are forms of labor, we did not expect the two constructs to be correlated because cognitive labor is a form of unpaid labor that is boundaryless and underpins various tasks and spans different domains (Daminger, 2019). Thus, cognitive labor is likely to occur at all times of the day, while one is engaged in other tasks, and, unlike other forms of labor such as household labor or childcare, it is not likely to take away from paid work or as likely to be influenced by paid work. As for social desirability and political skill, we are unaware of any reason or research indicating these should be related to division of cognitive labor.

Finally, to assess criterion-related validity, we examined the relationship between division of cognitive labor and perceived time poverty, emotional exhaustion, and relationship satisfaction. Perceived time poverty refers to the perception of lacking free time or time to engage in leisure activities (Zheng et al., 2022). Given that cognitive labor is an invisible and often constant form of labor, we expected that division of cognitive labor is positively related to perceived time poverty. Similarly, due to the ongoing and taxing nature of cognitive labor (Daminger, 2019), we expected that it is positively related to emotional exhaustion, which is defined as the depletion of psychological and emotional resources (Maslach & Jackson, 1981). Finally, given that division of cognitive labor represents a disproportionate amount of engagement, compared to one’s partner, in a form of labor that is invisible, constant, and taxing, we expected that in line with prior research (Reich-Stiebert et al., 2023), division of cognitive labor is negatively related to relationship satisfaction as it is likely related to perceptions of unfairness.

**Method**

**Participants and Procedure**

Participants were 275 individuals based in the United States (80%, *n* = 221) or Canada (20%, *n =* 54) who self-identified as men (50%, *n* = 137) or women (50%, *n =* 138). We recruited participants who are employed full time, in a heterosexual relationship with an employed partner, and living with their partner. The majority of our participants were married (75%, *n* = 206) and had children (67%, *n* = 185). Of those who have children, 80% (*n* = 147) had children under the age of 18 living with them. Participants self-identified as White (74%, *n* = 202), East Asian (6%, *n* = 16), Black/African American/Canadian (6%, *n* = 16), Hispanic/Latino (5%, *n* = 14), Southeast Asian (2%, *n* = 6), South Asian (2%, *n* = 6), First Nations/Indigenous (<1%, *n* = 1), Middle Eastern (<1%, *n* = 1), West Indian (<1%, *n* = 1), other (West Asian; <1%, *n* = 1), and multiracial (4%, *n* = 11). They were highly educated (72% had completed or were completing a four-year college or university degree or other postsecondary education, *n* = 197) and worked in various industries (e.g., education, healthcare, retail, manufacturing, information technology, finance). On average, participants were 42.28 years old (*SD* = 11.13) and had been employed at their current organization for 9.56 years (*SD* = 8.18).

A total of 301^[[1]](#footnote-1)^ participants were recruited from Prolific, an online crowdsourcing platform designed to recruit participants for scientific research (Peer et al., 2017). All respondents were compensated £2. The final sample size was *n* = 275 based on the criteria that they (a) answered at least two out of three attention checks correctly (Cheung et al., 2017) and (b) agreed to the use of their data. Participants completed an online survey in which the ostensible purpose of the study was to examine people’s current home and work experiences. They were asked to complete the measures described below. As with our main study, this study was reviewed and approved by the Institutional Review Board prior to data collection (protocol #6505; “Understanding couples’ work and home experiences”).

**Measures**

All participants completed the measures outlined below except for division of childcare, which was only completed by participants who indicated that they had a child under the age of 18 living with them. All measures, with the exception of the measures of social desirability and political skills, referred specifically to what occurred over the past week where participants reported on their feelings and behaviors and used a 7-point Likert response scale (1 = *my partner did nearly all of this* to 7 = *I did nearly all of this*) unless otherwise noted. A full list of measures can be found in Appendix A.

***Focal Variable***

We assessed *division of* *cognitive labor* with four items that drew upon Daminger’s (2019) qualitative work (e.g., “anticipating and recognizing upcoming needs, problems, or opportunities;” α = .90).

***Convergent Validity***

*Division of household labor* was measured with Mannino and Deutsch’s (2007) four-item scale (e.g., “shopping for groceries,”. α = .71). *Division of childcare* was measured with Mannino and Deutsch’s (2007) eight-item scale (e.g., “responding to your children’s requests and ongoing needs;” α = .88). *Division of emotion* *work* was measured using Erickson’s (1993) 12-item scale (e.g., “offer family members encouragement;” α = .94). *Division of prospective memory* was measured with Harrington and Reese-Melancon’s (2022) four-item scale (e.g., “making sure members of our household accomplish the things they need to do in a given day;” α = .95). The items of this scale were modified to refer to members of the household generally, as opposed to referring specifically to one’s child(ren), partner, or oneself.

***Discriminant Validity***

*Division of paid labor* was measured with two items that asked about household income (1 = *my partner brought in nearly all of our household income* to 7 = *I brought in nearly all of our household income*) and time worked (1 = *my partner spent much more time* *working* to 7 = *I spent much more time working*; α = .83) over the past week.

*Social desirability* was measured with Hart et al.’s (2015) 16-item balanced inventory of desirable responding – short form (overall α = .86). Both this scale and the political skill scale referred to how participants feel about themselves generally and used the following 7-point Likert response scale: 1 = *strongly disagree* to 7 = *strongly agree*. This measure can be broken down into two eight-item subscales: *impression management* (e.g., “I never regret my decisions;” α = .81) and *socially desirable responding* (e.g., “When I hear people talking privately, I avoid listening;” α = .82).^[[2]](#footnote-2)^

*Political skill* was measured using Ferris et al.’s (2005) 18-item scale (e.g., “I am good at building relationships with influential people at work;” α = .94).

***Criterion-Related Validity***

*Perceived time poverty* was measured using Zheng et al.’s (2022) six-item scale (e.g., “I often feel like there's no time for personal recreation;” α = .95; 1 = *not at all* to 7 = *completely*). *Emotional exhaustion* was measured using Maslach and Jackson’s (1986) nine-item measure (e.g., “I have felt used up at the end of the day;” α = .96; 1 = *not at all* to 7 = *a great extent*). *Relationship satisfaction* was measured using Funk and Rogge’s (2007) four-item scale (e.g., “How rewarding is your relationship with your partner?;” α = .95; 1 = *not at all* to 7 = *completely* for items 1-3; 1 = *extremely unhappy* to 7 = *perfect* for item 4).

**Results**

All data was analyzed using SPSS v.28.0.1.0.

**Exploratory Factor Analysis**

A principal axis factor analysis was conducted on the four items of the division of cognitive labor scale with oblique rotation (direct oblimin). The Kaiser-Meyer-Olkin measure verified the sampling adequacy for the analysis, KMO = .841. One factor was extracted with an eigenvalue over 1, explaining 68.76% of the variance. The scree plot also suggested one factor. Table S1 shows the factor loadings after rotation. All four items loaded strongly onto the underlying factor.

**Nomological Network**

Table S2 contains means, standard deviations, alpha coefficients, and bivariate correlations for the measures used within the study. The correlations suggest evidence supporting most of our expected relationships. Specifically, demonstrating convergent validity, division of cognitive labor was positively and significantly correlated with division of household labor (*r* = .22, *p* < .001), division of childcare (*r* = .23, *p* = .005), division of emotion work (*r* = .36, *p* < .001), and division of prospective memory (*r* = .41, *p* < .001).

However, evidence for discriminant validity was a little weaker. Division of cognitive labor was significantly correlated with division of paid labor (*r* = .13, *p* = .035), social desirability (*r* = .15, *p* = .014), and political skill (*r* = .20, *p* = .001). Specifically, it was significantly correlated with the impression management subscale of social desirability (*r* = .16, *p* = .007), but not the socially desirable responding subscale (*r* = .09, *p* = .119). Although these relationships were significant, they were generally modest (i.e., “small” effects, Cohen, 1992) and weaker than the predicted relationships (i.e., convergent validity estimates), such that the overall pattern with regard to strength of associations still generally conforms to expectations.

In terms of criterion-related validity, the division of cognitive labor was positively related to emotional exhaustion (*r* = .12, *p* = .046) and negatively related to relationship satisfaction (*r* = -.18, *p* = .003). This is in line with emerging research indicating that gendered mental load is negatively related to well-being and relationship satisfaction (Reich-Stiebert et al., 2023). However, contrary to expectations, division of cognitive labor was unrelated to perceived time poverty (*r* = .05, *p* = 375). Perhaps this relationship was not significant because cognitive labor tends to co-occur in between or alongside other activities or labor such that people may not notice that it consumes time in their minds or lives.

Given that the impression management subscale of social desirability was significantly correlated with division of cognitive labor, such that those higher on impression management tended to report that they engaged in more labor than their partners, we conducted regression analyses where we controlled for this variable to see if it affected the observed relationship between division of cognitive labor and theorized outcomes variables (i.e., perceived time poverty, emotional exhaustion, relationship satisfaction). Relationships between division of cognitive labor and both emotional exhaustion (*b* = .33, *SE* = .08, *p* < .001) and relationship satisfaction (*b* = -.29, *SE* = .07, *p* < .001) remained significant. In contrast, the relationship between division of cognitive labor and perceived time poverty was now significant (*b* = .19, *SE* = .09, *p* = .041). We speculate that perhaps this occurred because individuals higher on the impression management subscale of social desirability may be less likely to admit that they are experiencing time poverty as it may indicate their inability to manage their life responsibilities, such that this relationship only becomes apparent when we control for this individual difference.

**Discussion**

The results of this study provided some evidence of convergent, discriminant, and criterion-related validity of the division of cognitive labor measure used in the study in-text. Specifically, providing support for convergent validity, division of cognitive labor was found to be positively correlated with other forms of unpaid labor that involve caring for members of one’s household, including division of household labor, division of childcare, and division of emotion work. It was also found to be positively correlated with division of prospective memory, a form of ongoing mental labor similar to division of cognitive labor. Additionally, while division of cognitive labor was found to be positively correlated with division of paid labor, social desirability (in particular, the impression management dimension), and political skill, the relationships were generally modest (i.e., “small” effects, Cohen, 1992) and weaker than the convergent validity estimates. Thus, we offer that the overall nomological network that emerges with regard to strength of associations still generally conforms to our predictions and provides some evidence for discriminant validity.

Finally, providing support for criterion-related validity, division of cognitive labor was positively related to perceived time poverty (when controlling for the impression management subscale of the social desirability measure) and emotional exhaustion as well as negatively related to relationship satisfaction, although we acknowledge that these relationships are modest. These results suggest that this invisible and constant form of labor may serve as a drain on people’s time, energy, and relational resources.

**Table S1**

*Study Results from a Factor Analysis of the Division of Cognitive Labor Scale*

| Scale Items | | Factor Loadings |
| --- | --- | --- |
| 1. | Anticipating and recognizing upcoming needs, problems, or opportunities | .802 |
| 2. | Determining options for fulfilling needs | .876 |
| 3. | Choosing among previous identified options | .794 |
| 4. | Ensuring decisions were executed and needs sufficiently addressed | .842 |

.

**Table S2**

*Descriptive Statistics, Cronbach’s Alphas, and Zero Order Correlations*

| Variable | *M* | *SD* | 1 | 2 | 3 | 4 | 5 | 6 | 7 | 8 | 9 | 10 | 11 | 12 | 13 |
| --- | --- | --- | --- | --- | --- | --- | --- | --- | --- | --- | --- | --- | --- | --- | --- |
| 1. DOL_CL_ | 4.87 | 1.08 | (.90) |  |  |  |  |  |  |  |  |  |  |  |  |
| 2. DOL_HL_ | 4.62 | 1.39 | .22*** | (.71) |  |  |  |  |  |  |  |  |  |  |  |
| 3. DOL_CH_ | 4.44 | 1.11 | .23** | .59*** | (.88) |  |  |  |  |  |  |  |  |  |  |
| 4. DOL_EW_ | 4.42 | .84 | .36*** | .28*** | .56*** | (.94) |  |  |  |  |  |  |  |  |  |
| 5. DOL_PM_ | 4.64 | 1.18 | .41*** | .44*** | .68*** | .51*** | (.95) |  |  |  |  |  |  |  |  |
| 6. DOL_PL_ | 4.57 | 1.55 | .13* | -.31*** | -.37*** | -.04 | -.20*** | (.83) |  |  |  |  |  |  |  |
| 7. BIDR | 4.44 | .94 | .14** | -.08 | -.04 | .03 | -.02 | .12* | (.86) |  |  |  |  |  |  |
| 8. BIDR_IM_ | 4.42 | 1.05 | .16** | -.12* | -.14 | -.06 | -.06 | .18** | .85*** | (.81) |  |  |  |  |  |
| 9. BIDR_SDR_ | 4.46 | 1.14 | .09 | -.02 | .05 | .10 | .03 | .04 | .87*** | .48*** | (.82) |  |  |  |  |
| 10. PSI | 5.17 | .95 | .19** | -.02 | .01 | .12 | .08 | .16** | .39*** | .43*** | .26*** | (.94) |  |  |  |
| 11. PTP | 3.91 | 1.75 | .05 | .10 | .23** | .23*** | .15* | -.01 | -.34*** | -.37*** | -.23*** | -.14** | (.95) |  |  |
| 12. EE | 3.75 | 1.73 | .12* | .18** | .26** | .21*** | .22*** | -.10 | -.47*** | -.49*** | -.33*** | -.27*** | .62*** | (.96) |  |
| 13. RS | 5.72 | 1.35 | -.18** | -.14* | -.31*** | -.30*** | -.23*** | .01 | .29*** | .31*** | .19** | .28** | -.27*** | -.30*** | (.97) |

*Note. n* ranges from 147 (division of childcare measure) to 275. Cronbach’s alphas are presented in parentheses. DOL_CL_ = division of cognitive labor; DOL_HL_ = division of household labor; DOL_CH_ = division of childcare; DOL_EW_ = division of emotion work; DOL_PM_ = division of prospective memory; DOL_PL_ = division of paid labor; BIDR= behavioural inventory of desirable responding; BIDR_IM_ = impression management subscale of behavioural inventory of desirable responding; BIDR_SDR_ = socially desirable responding subscale of behavioural inventory of desirable responding; PSI = political skill inventory; PTP = perceived time poverty; EE = emotional exhaustion; RS = relationship satisfaction. *p < .05. **p < .01. ***p < .001.

**Appendix A**

**Validation Study Measures**

**Division of cognitive labor** (Daminger, 2019)

In the past week, between you and your partner, what was the typical distribution among the following? (1 = *my partner did nearly all of this*, 4 = *we were sharing this equally*, 7 = *I did nearly all of this*)

1. Anticipating and recognizing upcoming needs, problems, or opportunities.
2. Determining options for fulfilling needs.
3. Choosing among previously identified options.
4. Ensuring decision was executed and need sufficiently addressed.

**Division of household labor** (Mannino & Deutsch, 2007)

In the past week, between you and your partner, what was the typical distribution of effort among the following? (1 = *my partner did nearly all of this*, 4 = *we were sharing this equally*, 7 = *I did nearly all of this*)

1. Shopping for groceries
2. Cooking meals
3. Doing the dishes
4. Cleaning the house

**Division of childcare** (Mannino & Deutsch, 2007)

In the past week, between you and your partner, what was the typical distribution of effort among the following? (1 = *my partner did nearly all of this*, 4 = *we were sharing this equally*, 7 = *I did nearly all of this*)

1. Helping your children get dressed in the morning
2. Putting your children to bed
3. Playing with your children
4. Give your children a bath
5. Making decisions about your children’s upbringing
6. Reading to your children
7. Arranging for childcare
8. Responding to your children’s requests and ongoing needs

**Division of emotion work** (Erikson, 1993)

In the past week, between you and your partner, what was the typical distribution of effort among the following? (1 = *my partner did nearly all of this*, 4 = *we were sharing this equally*, 7 = *I did nearly all of this*)

1. Initiate talking things over with family members.
2. Try to bring family members out of feeling restlessness, boredom, or depression.
3. Let family members know I have faith in them.
4. Sense that family members are disturbed about something.
5. Offer family members encouragement.
6. Give family members compliments.
7. Stick by family members in times of trouble.
8. Offer family member advice when they are faced with a problem.
9. Respect family members’ point of view.
10. Act affectionately toward family members.
11. Express concern for family members’ well-being.
12. Do favors for family members without being asked.

**Division of prospective memory** (Harrington & Reese-Malancon, 2022)

Over the past week, between you and your spouse, what was the distribution of effort among each of the following? (1 = *my partner did nearly all of this*, 4 = *we were sharing this equally*, 7 = *I did nearly all of this*)

1. Helping the members of our household plan the things they need to do in a given day.
2. During the day, reminding members of our household of the things they need to do.
3. Making sure members of our household accomplish the things they need to do in a given day.
4. At some point during the day, asking members of our household whether they completed the tasks they needed to do that day.

**Division of paid labor**

Over the past week, between you and your partner:

1. What was the distribution of household income? (1 = *my partner brought in nearly all of our household income*, 4 = *we contributed equally to the household income*, 7 = *I brought in nearly all of our household income*)
2. What was the distribution of work hours? (1 = *my partner spent much more time working*, 4 = *we worked equal hours*, 7 = *I spent much more time working*)

**Balanced inventory desirable responding – short form** (i.e., social desirability; Hart et al., 2015)

Please rate the extent to which you agree with the following statements: (1 = *strongly disagree*, 7 = *strongly agree*)

*Impression management*

1. I have not always been honest with myself
2. I always know why I like things
3. It’s hard for me to shut off a disturbing thought
4. I never regret my decisions
5. I sometimes lose out on things because I can’t make up my mind soon enough
6. I am a completely rational person
7. I am very confident about my judgments
8. I have sometimes doubted my ability as a lover

*Socially desirable responding*

1. I sometimes tell lies if I have to
2. I never cover up my mistakes
3. There have been occasions when I have taken advantage of someone
4. I sometimes try to get even rather than forgive and forget
5. I have said something bad about a friend behind his or her back
6. When I hear people talking privately, I avoid listening
7. I never take things that don’t belong to me
8. I don’t gossip about other people’s business

**Political skill inventory** (Ferris et al., 2005)

Please rate the extent to which you agree with the following statements about yourself at work: (1 = *strongly disagree*, 7 = *strongly agree*)

*Networking ability:*

1. I spend a lot of time and effort at work networking with others.
2. At work, I know a lot of important people and am well connected.
3. I am good at using my connections and networks to make things happen at work.
4. I have developed a large network of colleagues and associates at work who I can call on for support when I really need to get things done.
5. I spend a lot of time at work developing connections with others.
6. I am good at building relationships with influential people at work.

*Apparent sincerity*

1. It is important that people believe I am sincere in what I say and do.
2. When communicating with others, I try to be genuine in what I say and do.
3. I try to show a genuine interest in other people.

*Social astuteness*

1. I always seem to instinctively know the right thing to say or do to influence others.
2. I have good intuition or savvy about how to present myself to others.
3. I am particularly good at sensing the motivations and hidden agendas of others.
4. I pay close attention to people’s facial expressions.
5. I understand people very well.

*Interpersonal influence*

1. It is easy for me to develop good rapport with most people.
2. I am able to make most people feel comfortable and at ease around me.
3. I am able to communicate easily and effectively with others.
4. I am good at getting people to like me.

**Perceived time poverty** (Zheng et al., 2022)

Please indicate the extent to which the following items describe how you have felt over the past week: (1 = *not at all* to 7 = *completely*)

1. I often feel like there’s no time for socializing.
2. I often feel like there's no time for personal recreation.
3. I often feel like there's no time for traveling.
4. I often feel like there's no time for exercising.
5. I often feel like there's no time to do the things that I love to do.
6. I often feel like I don't have time to get things done that I planned in my private life.

**Emotional exhaustion** (Maslach & Jackson, 1986)

Please rate the extent to which you have felt the following in the past week: (1 = *not at all* to 7 = *to a great extent*)

1. I have felt emotionally drained.
2. I have felt used up at the end of the day.
3. I have felt fatigued when I get up in the morning and have to face another day.
4. Being around people all day has really been a strain on me.
5. I have felt burnt out.
6. I have felt frustrated.
7. I have felt I’m working too hard.
8. Working with people has put too much stress on me.
9. I have felt like I’m at the end of my rope.

**Relationship satisfaction** (Funk & Rogge, 2007)

Please indicate the extent to which the following describes how you have felt over the past week: (1 = *not at all* to 7 = *completely*)

1. I have a warm and comfortable relationship with my partner
2. How rewarding is your relationship with your partner?
3. In general, how satisfied are you with your relationship?
4. Please indicate the degree of happiness, all things considered, in your relationship (1 = *extremely unhappy* to 7 = *perfect*)

**Results of Preliminary Analyses**

We examined whether division of labor was changing during the pandemic using latent growth models (in Mplus) to assess division of labor over the seven weeks of our study. Prior to conducting longitudinal analyses, we tested measurement variance to examine whether the measurements of division of cognitive, paid, and household labor, along with division of childcare were equivalent across measurement occasions. This is necessary to ensure that the same construct is being compared across time (Ployhart & Vandenberg, 2010).

We followed recent recommendations from Vandenberg and Morelli (2016) and tested configural invariance, which refers to equivalence in the pattern of factor loadings across occasion of measurement (i.e., items were specified as loading on a single factor that corresponded to measurement occasion). Results supported configural invariance for cognitive labor (χ^2^= 1250.90, *df* = 329, CFI = .87, TLI = .86, RMSEA = .10, SRMR = .05) and paid labor (χ^2^ = 59.14, *df* = 35, CFI = .99, TLI = .99, RMSEA = .05, SRMR = .02). We also tested metric invariance, which examines whether items’ factor loadings are the same across time (i.e., factor loadings were constrained to be equal across measurement occasions). Results supported metric invariance for cognitive labor (χ^2^ = 1272.78, *df* = 347, CFI = .10, TLI = .86, RMSEA = .10, SRMR = .05) and paid labor (χ^2^ = 59.14, *df* = 35, CFI = .99, TLI = .99, RMSEA = .05, SRMR = .02). Thus, we examined latent growth models for cognitive labor and paid labor (see results reported in-text).

By contrast, measurement invariance was not established for division of household labor or childcare. Specifically, results did not support configural invariance for household labor (χ^2^ = 5490.06, *df* = 329, CFI = .38, TLI = .29, RMSEA = .24, SRMR = .21) or childcare (χ^2^ = 2441.83,*df* = 1295, CFI = .86, TLI = .83, RMSEA = .09, SRMR = .07). Given that establishing configural invariance is a prerequisite for testing metric invariance, we did not test metric invariance. We speculate that one reason for this result could be because the aspects of household labor or childcare that were most salient or challenging changed over the course of the study, perhaps due to current events (e.g., variation in supply chain disruptions) or as a result of increased familiarity (e.g., more experience with supervising remote schooling).

**Results of Supplemental Analyses**

One concern that could be raised about our analytical approach is that our conceptual model is directional, implying a temporal sequencing that is not reflected in our main analyses. Therefore, we also conducted supplemental analyses based on time-separated measurements in which we estimate division of cognitive labor (as well as division of household labor, paid labor, and childcare) by averaging responses to weekly surveys 1, 2 and 3, emotional exhaustion by averaging responses to weekly surveys 4 and 5, and turnover intentions and career resilience by averaging responses to weekly surveys 6 and 7.

We first examined whether (a) average division of childcare from weekly surveys 1-3 and (b) average emotional exhaustion from weekly surveys 4-5 serially mediate the indirect effect of gender on average work outcomes (i.e., turnover intentions and career resilience) from weekly surveys 6-7 (controlling for average division of household and paid labor from weekly surveys 1-3). A bootstrapping procedure with 10,000 samples using PROCESS macro (Model 6; Hayes, 2022) in SPSS v.28.0.1.0 revealed that women (vs. men) reported engaging in a disproportionate amount of cognitive labor within their household in weeks 1-3 (*b* = .27, *SE* = .12, *p* = .022). Further, being responsible for a higher proportion of cognitive labor within one’s household was positively associated with emotional exhaustion in weeks 4-5 (*b* = .41, *SE* = .13, *p* = .002). In turn, emotional exhaustion was positively related to turnover intentions (*b* = .35, *SE* = .08, *p* < .001) and negatively related to career resilience (*b* = -.21, *SE* = .05, *p* < .001) in weeks 7-8. Further, bootstrapped tests of the indirect effects (10,000 iterations) revealed that the effect of gender on turnover intentions (indirect effect = .04, *SE* = .02, 95% CI [.005, .095]) and career resilience (indirect effect = –.02, *SE* *=* .01, 95% CI [-.057, -.003]) serially through (a) cognitive labor and (b) emotional exhaustion was significant.

Additionally, we tested whether (a) average division of childcare from weekly surveys 1-3 and (b) average emotional exhaustion from weekly surveys 4-5 serially mediate the indirect effect of gender on average work outcomes (i.e., turnover intentions and career resilience) from weekly surveys 6-7 for participants who indicated that they have a child under the age of 18 living with them (controlling for average division of cognitive, household, and paid labor from weekly surveys 1-3). We found that mothers (vs. fathers) reported engaging in a disproportionate amount of childcare within their household in weeks 1-3 (*b* = .83, *SE* = .13, *p* < .001). Being responsible for a higher proportion of childcare within one’s household was positively associated with emotional exhaustion in weeks 4-5 (*b* = .87, *SE* = .30, *p* = .004). In turn, emotional exhaustion was negatively related to career resilience (*b* = -.30, *SE* = .07, *p* < .001), but was not related to turnover intentions (*b* = .21, *SE* = .12, *p* = .074) in weeks 6-7. Further, tests of the indirect effects (10,000 iterations) revealed that the effect of gender on career resilience (indirect effect = -.22, *SE* *=* .10, 95% CI [-.433, -.060]) serially through (a) childcare and (b) emotional exhaustion was significant, but the effect on turnover intentions (indirect effect = .15, *SE* = .11, 95% CI [-.022, .416]) was not significant.

Overall, the pattern of results and conclusions were congruent with our hypotheses tests and exploratory analyses reported in-text. Namely, the results suggest that when women were unduly responsible for cognitive labor in their households for a period of time, they subsequently experienced greater emotional exhaustion, which then contributed to greater turnover intentions and lower career resilience at a later time. Further, for women with children, being unduly responsible for childcare for a period of time was positively related to subsequent emotional exhaustion, which then contributed to lower career resilience, but not greater turnover intentions, at a later time.

**References**

Ahearn, K. K., Ferris, G. R., Hochwarter, W. A., Douglas, C., & Ammeter, A. P. (2004). Leader political skill and team performance. *Journal of Management*, *30*(3), 309-327. https://doi.org/10.1016/j.jm.2003.01.004

Erickson, R. J. (1993). Reconceptualizing family work: The effect of emotion work on perceptions of marital quality. *Journal of Marriage and the Family, 55*(4), 888-900. https://doi.org/10.2307/352770

Ferris, G. R., Treadway, D. C., Kolodinsky, R. W., Hochwarter, W. A., Kacmar, C. J., Douglas, C., & Frink, D. D. (2005). Development and validation of the political skill inventory. *Journal of Management*, *31*(1), 126-152. https://doi.org/10.1177/0149206304271386

Funk, J. L., & Rogge, R. D. (2007). Testing the ruler with item response theory: Increasing precision of measurement for relationship satisfaction with the Couples Satisfaction Index. *Journal of Family Psychology*, *21*(4), 572-583. https://doi.org/10.1037/0893-3200.21.4.572

Hayes, A. F. (2022). *Introduction to Mediation, Moderation, and Conditional Process Analysis: A Regression-Based Approach* (Third edition). The Guilford Press.

Harrington, E. E., & Reese-Melancon, C. (2022). Who is responsible for remembering? Everyday prospective memory demands in parenthood. *Sex Roles,* *86*(3), 189–207. https://doi.org/10.1007/s11199-021-01264-z

Hart, C. M., Ritchie, T. D., Hepper, E. G., & Gebauer, J. E. (2015). The balanced inventory of desirable responding short form (BIDR-16). *Sage Open*, *5*(4), 1-9. https://doi.org/10.1177/2158244015621113.

Hinkin, T. R. (1998). A brief tutorial on the development of measures for use in survey questionnaires. *Organizational Research Methods*, *1*(1), 104-121. https://doi.org/10.1177/1094428198001001

Paulhus, D. L. (2002). Socially desirable responding: The evolution of a construct. In H. I. Braun, D. N. Jackson, & D. E. Wiley (Eds.), *The role of constructs in psychological and educational measurement* (pp. 49–69). Lawrence Erlbaum Associates Publishers.

Vandenberg, R. J., & Morelli, N. A. (2016). A contemporary update on testing for measurement equivalence and invariance. In *Handbook of employee commitment* (pp. 449-461). Edward Elgar Publishing.

Zheng, X., Zhang, Q., Li, X., & Wu, B. (2022). Being busy, feeling poor: The scale development and validation of perceived time poverty. *International Journal of Selection and Assessment*, *30*(4), 596-613. https://doi.org/10.1111/ijsa.12395

1. We recruited 300 participants. However, one participant completed the survey but was ‘timed out’ of Prolific; that is, it took them longer to complete the survey than Prolific expected them to so their submission was not recorded as one of the 300. Given they had completed the full survey, we compensated them. [↑](#footnote-ref-1)
2. We did not specify in our pre-registration that we may be examining the effects using the two social desirability subscales. This was an oversight and any analyses using the two subscales are exploratory. [↑](#footnote-ref-2)
